# 11β-HSD1 participates in epileptogenesis and the associated cognitive impairment by inhibiting apoptosis in mice

**DOI:** 10.1186/s12967-022-03618-x

**Published:** 2022-09-05

**Authors:** Xueying Li, Wanhua Qiu, Lu Deng, Jingjing Lin, Wenting Huang, Yuchen Xu, Mulan Zhang, Nigel C. Jones, Runxuan Lin, Huiqin Xu, Li Lin, Peijun Li, Xinshi Wang

**Affiliations:** 1 Department of Neurology, The First Affiliated Hospital of Wenzhou Medical University, Shangcai Village, Ouhai District, Wenzhou, Zhejiang Province People’s Republic of China; 2 School of Pharmaceutical Sciences, Wenzhou Medical University, Wenzhou, 325035 Zhejiang Province People’s Republic of China; 3 Department of Neurology, The Second Affiliated Hospital and Yuying Children’s Hospital of Wenzhou Medical University, Wenzhou, Zhejiang Province 325000 People’s Republic of China; 4 Department of Neuroscience, Central Clinical School, Monash University, Melbourne, VIC 2004 Australia; 5 Department of Neurology, The Alfred Hospital, Commercial Road, Melbourne, VIC 3004 Australia; 6 Department of Medicine, Royal Melbourne Hospital, The University of Melbourne, Melbourne, VIC 3052 Australia; 7 Department of Neurosurgery, First Affiliated Hospital of Wenzhou Medical University, Wenzhou, 325035 Zhejiang China; 8 Key Laboratory of Alzheimer’s Disease of Zhejiang Province, Institute of Aging, Wenzhou Medical University, Wenzhou, Zhejiang Province People’s Republic of China

**Keywords:** 11β-hydroxysteroid dehydrogenase 1 (11β-HSD1), Epilepsy, Cognitive impairment, Patch clamp, Apoptosis

## Abstract

**Background:**

Glucocorticoid signalling is closely related to both epilepsy and associated cognitive impairment, possibly through mechanisms involving neuronal apoptosis. As a critical enzyme for glucocorticoid action, the role of 11β-hydroxysteroid dehydrogenase 1 (11β-HSD1) in epileptogenesis and associated cognitive impairment has not previously been studied.

**Methods:**

We first investigated the expression of 11β-HSD1 in the pentylenetetrazole (PTZ) kindling mouse model of epilepsy. We then observed the effect of overexpressing 11β-HSD1 on the excitability of primary cultured neurons in vitro using whole-cell patch clamp recordings. Further, we assessed the effects of adeno-associated virus (AAV)-induced hippocampal 11β-HSD1 knockdown in the PTZ model, conducting behavioural observations of seizures, assessment of spatial learning and memory using the Morris water maze, and biochemical and histopathological analyses.

**Results:**

We found that 11β-HSD1 was primarily expressed in neurons but not astrocytes, and its expression was significantly (p < 0.05) increased in the hippocampus of PTZ epilepsy mice compared to sham controls. Whole-cell patch clamp recordings showed that overexpression of 11β-HSD1 significantly decreased the threshold voltage while increasing the frequency of action potential firing in cultured hippocampal neurons. Hippocampal knockdown of 11β-HSD1 significantly reduced the severity score of PTZ seizures and increased the latent period required to reach the fully kindled state compared to control knockdown. Knockdown of 11β-HSD1 also significantly mitigated the impairment of spatial learning and memory, attenuated hippocampal neuronal damage and increased the ratio of Bcl-2/Bax, while decreasing the expression of cleaved caspase-3.

**Conclusions:**

11β-HSD1 participates in the pathogenesis of both epilepsy and the associated cognitive impairment by elevating neuronal excitability and contributing to apoptosis and subsequent hippocampal neuronal damage. Inhibition of 11β-HSD1, therefore, represents a promising strategy to treat epilepsy and cognitive comorbidity.

## Introduction

Epilepsy is a common neurological disorder characterised by recurrent seizures. In addition to seizures, it is associated with a range of psychological, cognitive and behavioural disorders [1], which are critical determinants of impaired quality of life [2]. Cognitive impairment in epilepsy appears to be the consequence of complex interactions among the aetiologies of epilepsy, the severity of seizures themselves, interictal epileptiform discharges, and anti-epileptic drugs [3]. However, there is evidence that some patients have pre-existing cognitive complaints prior to the onset of epilepsy, the severity of which can predict treatment response [4]. These raise the necessity of novel therapeutic approaches which suppress seizures and also improve the associated cognitive impairment.

Stress is a commonly reported precipitant for seizures [5]. It is well documented that approximately half of individuals with epilepsy report more seizures following acute stressful situations or periods of stress [5]. As major stress hormones, the relationship between glucocorticoids and epilepsy is bidirectional. On the one hand, seizures and epilepsy can lead to activation of the hypothalamic–pituitary–adrenal (HPA) axis [6]; on the other hand, excessive glucocorticoids occurring in times of stress can contribute to epileptogenesis by increasing neuronal excitability and lowering seizure threshold in many animal models of epilepsy [7, 8]. In addition, hypersecretion of glucocorticoids can impair cognitive functioning [9]. As a key brain region involved in both epilepsy and cognition, the hippocampus contains a high density of glucocorticoid receptors [10] and this region is therefore particularly vulnerable to the detrimental effects of glucocorticoids [11]. For example, it was reported that aged people with significantly prolonged elevated cortisol level showed reduced hippocampal volumes and deficits in hippocampus-dependent cognitive functions compared to controls with normal cortisol levels [12]. Therefore, treatment targeting excessive glucocorticoid effects may be beneficial for both seizures and concomitant cognitive impairment.

Glucocorticoid activity is determined by the density of 'nuclear' receptors and intracellular metabolism by 11β-hydroxysteroid dehydrogenase (11β-HSD) enzymes, which act as intracellular gate-keepers of tissue glucocorticoid action [13]. Of these, 11β hydroxysteroid dehydrogenase 2 converts active glucocorticoids into inactive forms, and is mainly expressed in the target organs of mineralocorticoids, such as the kidney. In contrast, 11β-HSD1, found predominantly in the liver, adipose tissue and brain, catalyses circulating inert cortisone to active cortisol in humans (11β-dehydrocorticosterone to corticosterone in mice), and thus stimulates glucocorticoid action in these tissues [14]. In the brain, 11β-HSD1 is highly expressed in regions that underpin cognitive functions, including the prefrontal cortex and hippocampus [15]. Previous studies have shown that pharmacological inhibition or genetic knockdown of 11β-HSD1 protects against the cognitive impairment caused by excessive local steroid action in animal models of Alzheimer's disease and chronic stress [16, 17]. Apoptosis of neurons has been suggested to be an important mechanism underlying hippocampal neuronal death induced by glucocorticoids, leading to cognitive impairment [18, 19], and a previous study demonstrated that overexpression of 11β-HSD1 decreased cell proliferation and caused cell apoptosis [20].

Therefore, we hypothesise that 11β-HSD1 might participate in epileptogenesis, and that reducing glucocorticoid action in the brain by inhibiting 11β-HSD1 might suppress epileptic seizures, inhibit neuronal apoptosis induced by seizures, and protect against secondary cognitive impairment.

## Methods

### Animals

Forty adult male C57BL/6 mice (The Experimental Animal Center of Wenzhou Medical University, Zhejiang, China) weighing 20–25 g were used in this experiment. All mice were housed under controlled lighting conditions under 12/12 h light/dark cycle at a temperature of 22–26 °C, with water and food available ad libitum*.* Animals were housed for at least one week for acclimatisation before the stereotaxic procedure. All animals were used in compliance with the Institutional Review Board of Wenzhou Medical University (ethical batch number: 2021-0155), and experiments were performed based on the guideline of the National Institutes of Health for the care and use of laboratory animals.

### Primary cultures of mouse hippocampal neurons

Hippocampal neurons were prepared from C57BL/6 mice in accordance with established methodology [21]. Briefly, pregnant mice (E18) were sacrificed using CO_2_ euthanasia followed by decapitation, and the embryos were removed and maintained in HBBS. Hippocampus tissue was isolated from the embryos, and digested with 0.125% Trypsin–EDTA (Gibco) for 25 min at 37 °C after being minced into small pieces. The tissue pieces were then mechanically ground and separated in 2 ml DMEM (meilunbio) containing 10% FBS (Sigma) using a sterile, flame-polished glass Pasteur pipette. The cell suspension was centrifuged at 2000 rpm for 5 min, and the cell pellet was resuspended to obtain a concentration of 2.5 × 10^5^ cells/ml. Cells were then seeded on glass slides coated with Poly-l-Orithine (Sigma) in a 24-well plate and incubated for 4 h at 37 °C. After that, DMEM was discarded, and Neurobasal media supplemented with B27 (Gibco), and 0.5 mM glutamine (Gibco) was added.

### Construction of 11β-HSD1 overexpression plasmid and transfection of neurons

11β-HSD1 overexpression plasmid was constructed with a eukaryotic expression vector GV658 (CMV enhancer-MCS-polyA-EF1A-zsGreen-sv40-puromycin) encoding the amplified sequence of 11β-HSD1 (NM_008288). This was manufactured by Shanghai GeneChem Co., Ltd. (Shanghai, China).

Neurons cultured in vitro for three days (DIV3) were then transfected with the 11β-HSD1 overexpression plasmid. Briefly, 0.2 μg of plasmid diluted in 15 μl of Opti-MEM (Gibco) was mixed with 0.5 μl of Lipofectamine 2000 (Invitrogen) diluted in 15 μl Opti-MEM, and incubated for 25 min at room temperature to form DNA-Lipofectamine complexes. Next, about 30 μl of the complexes were added directly to each well containing cells, mixed gently and incubated at 37 °C for 48 h.

### Electrophysiological recordings

Hippocampal neurons were recorded by whole-cell patch clamp during continuous perfusion of artificial cerebrospinal fluid (aCSF, 2 mL/minute) at room temperature. Glass pipettes (non-filament, Garner Glass Company) were pulled (Model P-97, Sutter Instruments) to obtain electrodes with resistances between 5 and 7 MΩ when filled with intracellular solution (in mM:130 K-gluconate, 10 KCl, 2 MgCl_2_, 10 HEPES, 10 EGTA, 2 Na_2_-ATP, 0.2 Na_2_-GTP. pH 7.2, adjusted with KOH). Electrophysiological data were recorded in a whole cell configuration, a gigabit ohm seal was formed between the cell and the glass pipette, and then brief suction was used to break the cell membrane. Action potential (AP) threshold and firing frequency were recorded under current-clamp mode (Multiclamp 700A, molecular device). Digitisation (DigiData 1322, molecular device) was used for quick access to original traces, and all measures were offline analysed with PClamp10.6 software.

### Pentylenetetrazole (PTZ) kindling model

Mice were intraperitoneally injected with PTZ (35 mg/kg, Sigma, St. Louis, MO, USA) once every other day for a total of 14 injections (from day 1 to day 28). In contrast, control mice received vehicle (saline) injections. We observed the behaviour of each mouse for 1 h after PTZ injection to assess the resultant seizure severity, as judged using the Racine scale (1972) [22]. Seizure stages were classified: stage 0, no response; stage I, ear and facial twitching; stage II, myoclonic jerks (MJs); stage III, clonic forelimb convulsions; stage IV, generalised clonic seizures with turning to a side position; and stage V, generalised tonic–clonic seizures (GTCSs) or death. Mice with at least three consecutive seizures of stage IV or V were regarded as fully kindled.

### Virus construction and preparation

11β-HSD1 recombinant AAV expression vectors, along with the transgene for green fluorescent protein (GFP), were manufactured by Shanghai GeneChem Co., Ltd. (Shanghai, China). A universal scrambled sequence with mismatched bases was used as the negative control. The control shRNA targeting sequence is 5′-CGCTGAGTACTTCGAAATGTC-3′; the sequence of 11β-HSD1-shRNA was 5′-CCTGGCCTACTACTACTAT-3′. The shRNA target sequences were inserted into the GV478 lentivector. The GV478 lentivectors containing the shRNA sequences were transfected into 293 T cells, and viral supernatants were harvested after 48 h. The final virus titer was 2.02E + 12 v.g./ml.

### Intrahippocampal injections and grouping

Mice were anesthetised with 1% pentobarbital sodium (40 mg/kg, i.p.) and positioned in the stereotaxic apparatus. Mice were injected with AAV-11β-HSD1 (11β-HSD1-shRNA group) and AAV-blank (Con-shRNA group) bilaterally into the hippocampus as previously described [23]. Briefly, using the stereotaxic guidance, a micropipette was gently positioned in the hippocampus (bregma, − 2.2 mm; lateral, ± 2.2 mm; ventral, − 1.8 mm), and the shRNA was slowly infused into the region. The micropipette was held in place for an additional 10 min before being slowly withdrawn, and the incision was closed with sutures. Body temperature was maintained at 37 °C using a heating pad. To assess the efficiency of AAV-mediated knockdown, we randomly chose four mice from each group and sacrificed them on day 28 after AAV injection. The hippocampus was immediately isolated and prepared for either confocal scanning microscopy detection or western blot analysis. The remaining mice were intraperitoneally injected with PTZ from day 28 onwards to construct the epilepsy model described above.

To examine the expression of 11β-HSD1 in the hippocampus of epileptic mice, 20 mice were randomly subjected to intraperitoneal injection of PTZ or saline as a control, with 10 mice in each group. To investigate the effect of 11β-HSD1 knockdown on epileptogenesis and associated cognitive impairment, mice were divided into four groups: (1) the Control group (*n* = 10, mice received Saline injection only); (2) the Epilepsy group (*n* = 10, mice received PTZ (35 mg/kg) injection only); (3) the Con-shRNA group (*n* = 10, mice received successive injections of AAV-blank vectors and PTZ); and (4) the 11β-HSD1-shRNA group (*n* = 10, mice received successive injections of AAV-11β-HSD1 and PTZ).

### Morris water maze

After PTZ kindling, all mice were subjected to a Morris water maze (MWM) to evaluate spatial learning and memory. The maze consisted of a stainless-steel circular tank filled with water (22 ℃), divided into four virtual quadrants, and placed in a room with external visual cues. A hidden platform was submerged in one of the quadrants (kept constant for each mouse), ~ 1.5 cm below the water's surface. A camera was located above the centre of the maze that relayed images to a videocassette recorder and an image analysis computer system (DigBehv, Jiliang Software Technology Company, Shanghai, China). Mice went through a series of trials, where they were placed in the pool, and attempted to locate the hidden platform. The mice were allowed to swim for a period of 60 s to find the hidden platform, but if they failed to do so during this time, they were guided and placed on the platform for 15 s. Mice conducted four trials per session with an intertrial interval of 30 min, with each trial starting at a different point, alternating amongst the four quadrants. Sessions were conducted on five consecutive days. For each trial, we recorded the time that mice spent trying to locate the hidden platform and the swim length and speed. On the sixth day, the last day of experiment, we removed the platform and allowed the mice to swim freely for 60 s. We then recorded the number of crossings through the zone which previously held the target platform for each mouse.

### Immunofluorescence

On the 28th day after AAV injection, mice were anaesthetised and perfused through the left cardiac ventricle, the brains removed and postfixed overnight in 4% paraformaldehyde. Frozen sections were prepared using a cryostat microtome with a thickness of 7 μm. Next, the tissue sections were permeabilised with 0.25% Triton X-100 in PBS for 10 min. The tissue sections were washed three times with PBS before being blocked with 1% BSA for 30 min. After being rinsed with PBS, the tissue sections were incubated with anti-GFAP(1:50) and anti-11β-HSD1(1:50), or anti-NeuN(1:50) and anti-11β-HSD1(1:50) antibodies at 4 ℃ overnight. After being washed three times with PBS, the samples were incubated with secondary antibodies for 1 h at RT. After staining the nucleus with DAPI, images were captured using a confocal laser scanning microscope (A1tR, Nikon, Tokyo, Japan).

### Western blotting

Four mice were decapitated under 1% pentobarbital sodium, and their brains were collected immediately. Fresh hippocampal tissue samples were a random subset picked blindly. Tissue and cells were homogenised in lysis buffer with protease inhibitor mixture and were centrifuged at 12,000×*g* for 15 min, and then the supernatants were collected. Proteins were separated by SDS-PAGE gel (10% separation gel) and transferred to a PVDF membrane (Merck & Co., Inc., Whitehouse Station, NJ, USA, Germany). The membrane was blocked with 5% fat-free milk solution for 1 h at room temperature and incubated with respective primary antibodies overnight at 4 °C: 11β-HSD1 (ab169785, 1:1000, Abcam, Cambridge, United Kingdom), BCL-2 (#3498, 1:1000; Cell Signaling Technology), BAX (#14796, 1:1000; Cell Signaling Technology), Cleaved CASPASE-3 (#9661, 1:1000; Cell Signaling Technology) and GAPDH (#5174, 1:1000; Cell Signaling Technology). After being washed with TBST three times, immunoreactive bands were incubated with horseradish peroxidase (HRP) conjugated goat anti-rabbit secondary antibody (A0208, 1:2000; Beyotime) or goat anti-mouse secondary antibody (#SSA007, 1:5000, Sino biological). The ECL procedure (Bio-Rad) was used to detect the proteins.

### Nissl staining

Nissl staining was employed to detect surviving neurons. Two mice of hippocampal samples were embedded in paraffin and cut into 7 μm sections, and the sections were dewaxed and rehydrated according to the standard protocols. Next, the sections were stained in 1% cresyl violet at 50 ℃ for 5 min. After being rinsed with water, the sections were dehydrated in ethanol with increasing concentrations and mounted on the slides. The stained sections were then viewed under a microscope (Olympus Corporation, Tokyo, Japan).

### Immunohistochemistry

Fresh tissue was fixed in 4% paraformaldehyde and embedded in paraffin. Five-micron sections were obtained, deparaffinised, and rehydrated as previously described. After antigen retrieval, endogenous peroxidase was blocked using 3% hydrogen peroxide at room temperature for 10 min. Sections were blocked with 5% BSA and then incubated with primary antibody (11β-HSD1, 1:100, Abcam, Cambridge, United Kingdom) in a humid chamber at 4 °C overnight, followed by incubation with an HRP-conjugated secondary antibody (1:200) at room temperature for 1 h. After colour development through incubation with diaminobenzidine, the sections were counterstained with hematoxylin. The developed tissue sections were visualised under a microscope (Olympus Corporation).

### Statistical analysis

Statistical analysis was conducted using Graph-Pad Prism Version 8.0 for Windows (Graph-Pad Software, USA). All measurements of electrophysiological recordings were analysed offline using Clampfit software (v. 10.6 Molecular Devices). As previously described, we followed the methodology for obtaining electrophysiological parameters for active and passive membrane properties [24]. Statistical differences were determined using student t test or one-way analysis of variance with LSD post-hoc comparisons as appropriate. A p-value of less than 0.05 was considered statistically significant. Data were expressed as the mean ± standard deviation (SD).

## Results

### Neuronal expression of 11β-HSD1 is increased in the hippocampus of epilepsy mice

**Fig. 1:**
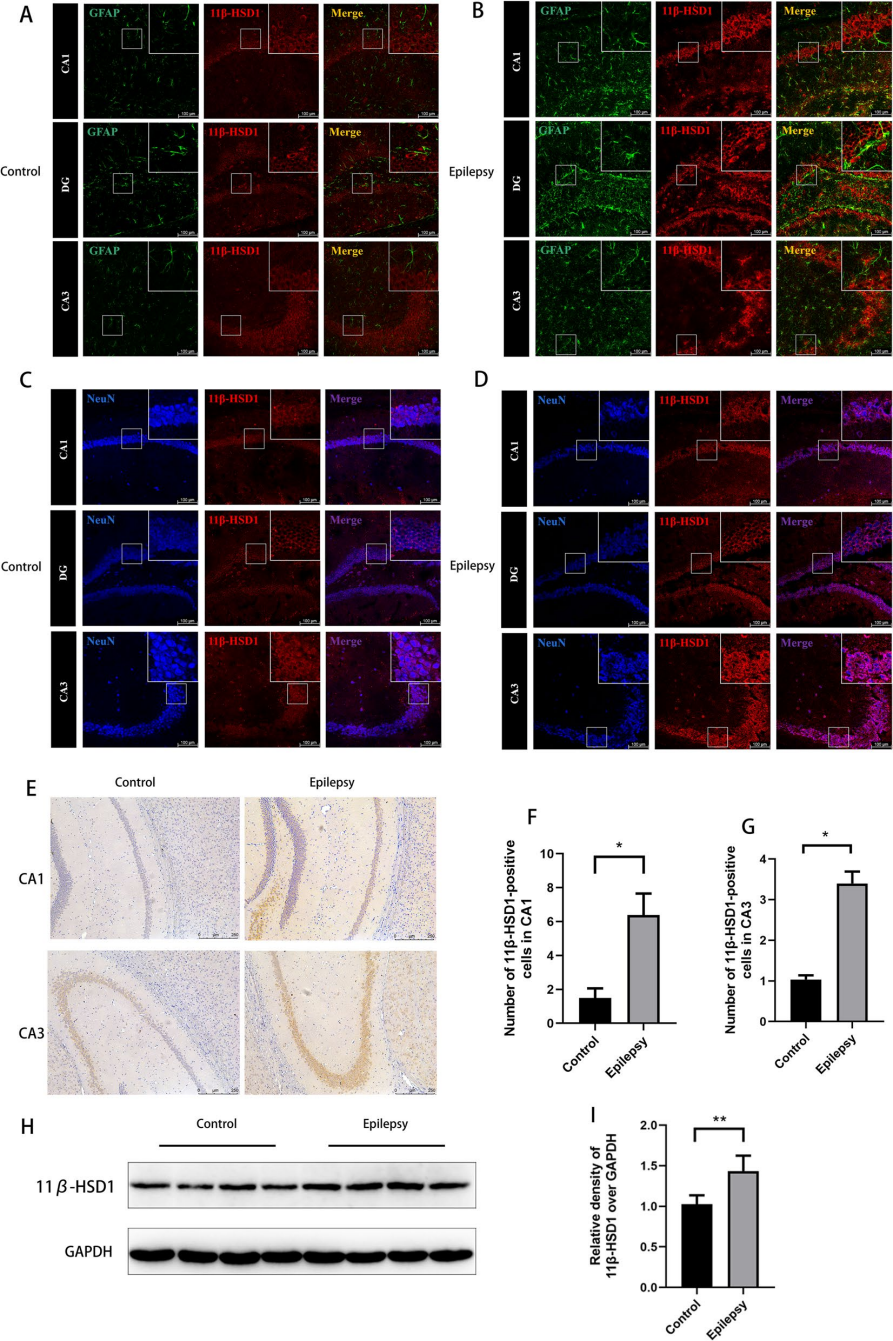
The expression of 11β-HSD1 increased in the hippocampus of epilepsy mice. **A**–**D** Representative images of 11β-HSD1 immunofluorescence. **E** Representative images of 11β-HSD1 immunohistochemical. **F**–**G** Statistical results showing the number of 11β-HSD1-positive cells in the CA1 and CA3 regions in each group. **H** The representative Western blot images show 11β-HSD1 expression in the hippocampus of control mice or epilepsy mice. **I** Statistical analysis of the intensity of 11β-HSD1 proteins relative to GAPDH (*P < 0.01, compared to control group). The bars indicated the mean ± SD

To explore the role of 11β-HSD1 in epilepsy, we first observed its expression in mice subjected to PTZ kindling. Immunofluorescence staining showed significantly increased expression of 11β-HSD1 (red) in the CA1, DG and CA3 regions of the hippocampus in the epilepsy mice compared with the control group. 11β-HSD1 mainly colocalised with NeuN (a neuron marker, blue) rather than GFAP (an astrocyte marker, green) (Fig. 1A–D), indicating 11β-HSD1 was primarily expressed in neurons rather than astrocytes. Immunohistochemistry staining also showed that the expression of 11β-HSD1 was significantly increased in the CA1 and CA3 regions of the hippocampus in the epilepsy mice (p < 0.01, Fig. 1E–G). Western blot analysis further confirmed that the expression of 11β-HSD1 protein was significantly increased in the hippocampus of PTZ-induced epilepsy mice compared to controls (p < 0.01, Fig. 1H, I).

### Overexpression of 11β-HSD1 elevates neuronal excitability in vitro

**Fig. 2:**
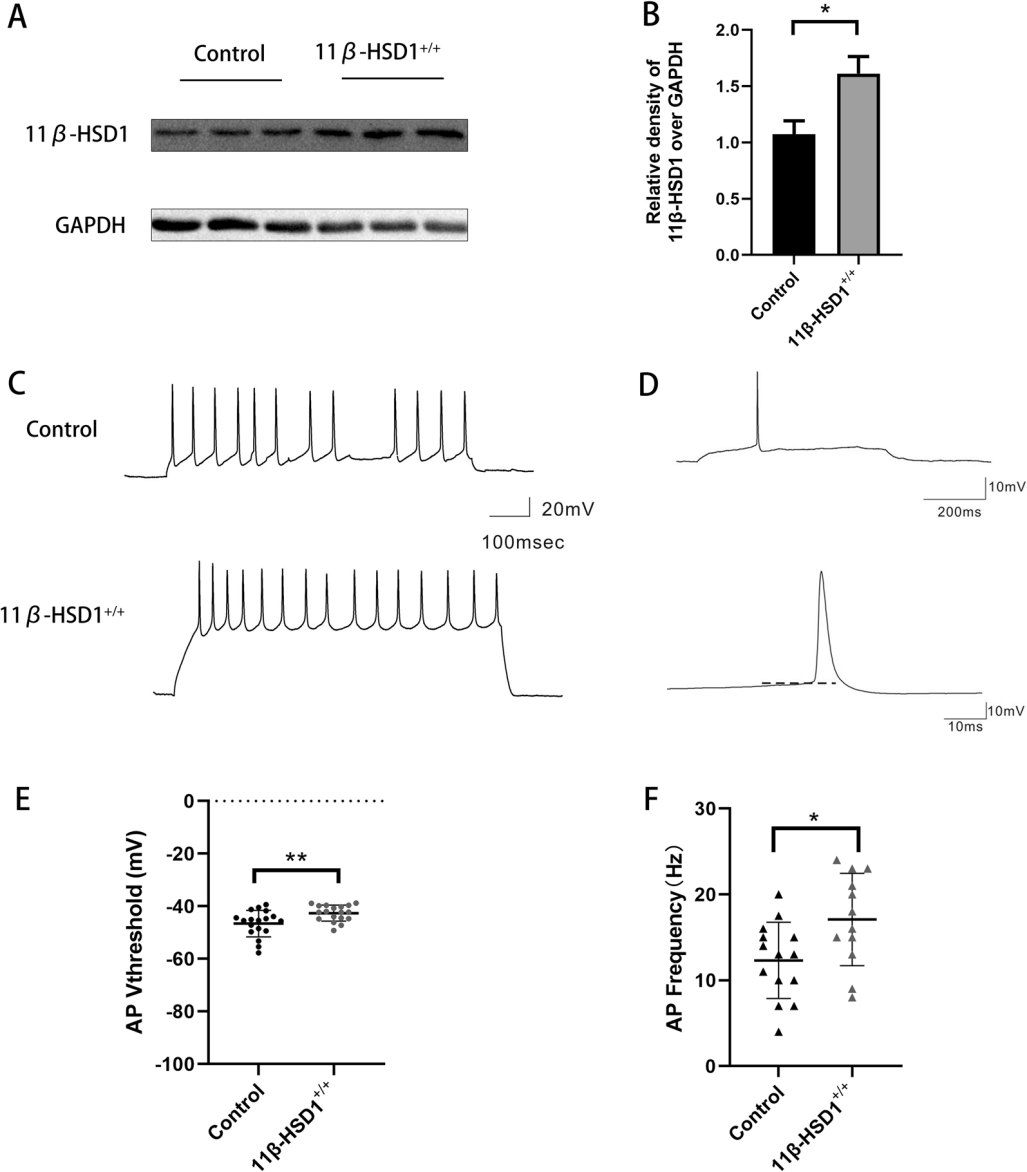
Overexpression of 11β-HSD1 elevated neuronal excitability in vitro. **A**, **B** Western blot images showing 11β-HSD1 expression in the primary cultured neurons transfected with 11β-HSD1 overexpression plasmid (11β-HSD1+/+) or control plasmid. **C** Raw trace of an action potential at depolarization recorded under current clamp mode. The lower panel shows an expanded image of the same action potential, with a dotted line demonstrating the action potential threshold. **D** Representative images of the APs recorded by patch clamp technique. **E**, **F** Statistical analysis of the threshold voltage and frequency of APs (*P < 0.01, compared to control group). The bars indicated the mean ± SD

To examine how increased expression of 11β-HSD1 may directly influence neuronal excitability, we used whole-cell patch clamping to examine the effect of 11β-HSD1 overexpression on the excitability of primary cultured hippocampal neurons by using an 11β-HSD1 overexpression plasmid (11β-HSD1+/+). First, we verified that our plasmid did indeed elevate the expression of 11β-HSD1 via Western Blot (Fig. 2A, B), compared to control. As shown in Table 1, the resistance and voltage of resting membrane potential (Rm and Vm) did not significantly vary between the two groups, although Vm displayed a tendency to increase in 11β-HSD1+/+ treated neurons. However, the threshold voltage to trigger an action potential was significantly decreased, while the frequency of action potentials increased in the 11β-HSD1+/+ neurons compared to controls (Fig. 2C–F). These results indicated that 11β-HSD1 overexpression increases neuronal excitability, and thus might contribute to epileptogenesis.

**Table 1:** Active and passive membrane properties of hippocampal neuron

	Control group (n = 18)	11β-HSD1^+/+^ group (n = 18)	P value
Membrane resistance (Ω)	432.18 ± 194.73	469.72 ± 162.48	0.5391
Membrane voltage (mV)	− 46.78 ± 12.14	− 38.56 ± 13.28	0.0607
AP threshold (mV)	− 42.61 ± 3.03	− 46.57 ± 5.02	**0.0070**
AP peak value (mV)	62.41 ± 14.47	64.55 ± 9.81	0.6078
AP Frequency (mV)	12.32 ± 4.44	17.08 ± 5.37	**0.021**

Significance (in bold) was determined by unpaired Student’s t-test, n = cell number;

AP, action potential

### 11β-HSD1 knockdown exerts anticonvulsant actions in PTZ-induced epilepsy mice

**Fig. 3:**
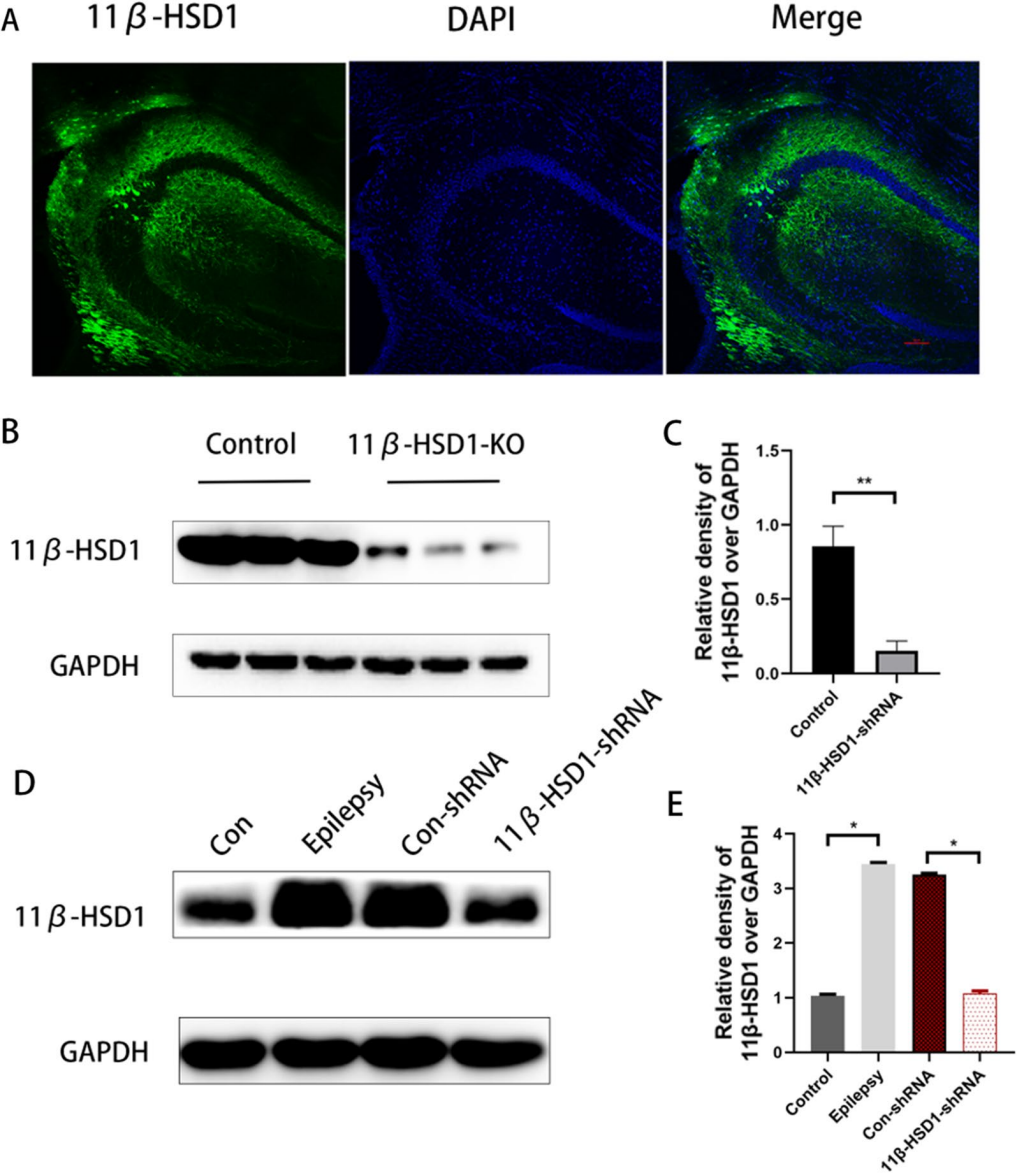
11β-HSD1 knockdown exerted anticonvulsant profile in PTZ-induced epilepsy mice. **A** Images showing GFP expression in the hippocampus four weeks after injection of recombinant AAV (cell nuclei were counterstained with DAPI). The scale bar = 200 μm. **B**, **C** Western blot images showing 11β-HSD1 expression in PTZ kindling mice intra-hippocampal injected with recombinant AAV containing 11β-HSD1 shRNA or control shRNA. **D**, **E** Western blot images showing 11β-HSD1 expression in the hippocampus of the control group, the epilepsy group, the Con-shRNA group and the 11β-HSD1-shRNA group (*P < 0.01, epilepsy group vs control group, Con-shRNA group vs 11β-HSD1-shRNA group). The bars indicated the mean ± SD

Thereafter, in order to test whether downregulation of 11β-HSD1 has an anti-epileptic effect, we developed a hippocampal 11β-HSD1-knockdown mouse using AAV technology prior to PTZ kindling. On the 28th day after AAV injection, the AAV was expressed throughout the hippocampus (Fig. 3A), and western blotting showed that the expression of 11β-HSD1 was significantly decreased compared to the control group (p < 0.01, Fig. 3B, C), indicating successful hippocampal knockdown of 11β-HSD1 protein.

**Fig. 4:**
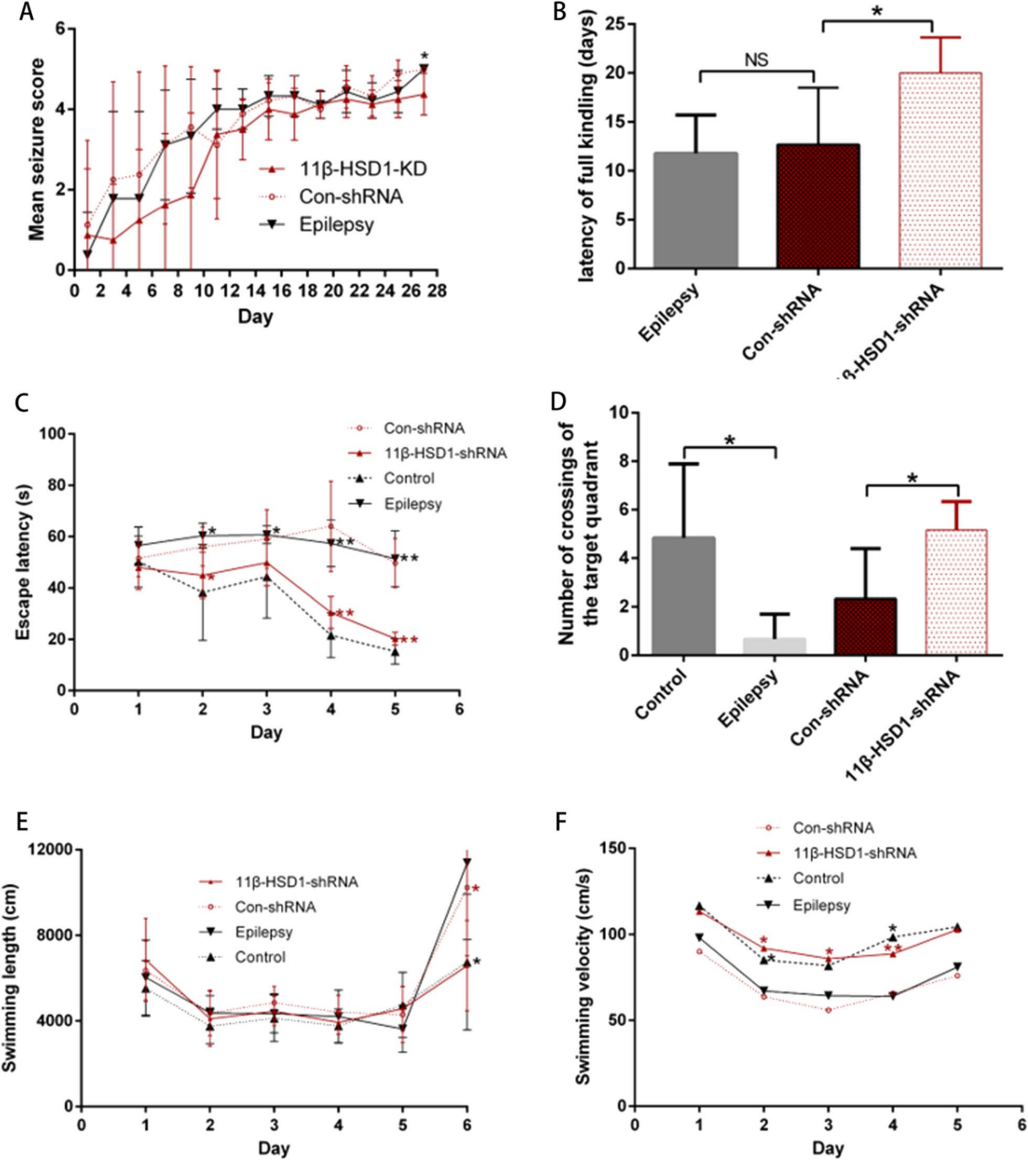
11β-HSD1 knockdown alleviated spatial learning and memory deficits in PTZ-induced epilepsy mice. **A**, **B** 11β-HSD1 knockdown significantly decreased seizure score and prolonged the number of days (latent period) required to reach complete kindling (n = 8 for the epilepsy and the Con-shRNA groups; n = 9 for the Con-shRNA and the 11β-HSD1-shRNA groups). **C**–**F** 11β-HSD1 knockdown significantly decreased escape latency, number of crossing of the target quadrant, swimming length and swimming velocity. (*P < 0.05, epilepsy group vs control group, Con-shRNA group vs 11β-HSD1-shRNA group). The bars indicated the mean ± SD

After developing this model, we first investigated the anticonvulsant effect of 11β-HSD1 knockdown. Seizure severity scores assessed by Racine scale, and the latency to achieve full kindling, were recorded. The mean seizure severity scores and the latency were not significantly different between the epilepsy group and the Con-shRNA group, indicating that the virus vector exerts no effect on PTZ kindling. However, there was a significant increase in the latency to full kindling in the 11β-HSD1-shRNA group compared to the Con-shRNA group (p = 0.021, Fig. 4B). Although the mean seizure severity score was not significantly varied in days 1–27 of kindling, 11β-HSD1-shRNA treated mice displayed a trend of reduced seizure severity, which reached statistical significance on the last day (p < 0.01, Fig. 4A), compared to Con-shRNA. Following bilateral hippocampal injection, two mice injected with AAV (one with 11β-HSD1-shRNA and one with Con-shRNA) failed to survive, presumably because of the excessive anesthetic dose. Two additional mice were added as replacements to maintain the same total number of animals in the experimental groups. Following intraperitoneal injections of PTZ, 80% of the mice in the epilepsy group without AAV injection and the Con-shRNA groups survived, while 90% of the mice in the Control group (without PTZ or AAV injection) and the 11β-HSD1-shRNA groups were alive. We finally had eight animals for the epilepsy and Con-shRNA groups, and 9 for the Control and 11β-HSD1-shRNA groups.

### 11β-HSD1 knockdown alleviates spatial learning and memory deficits in PTZ-induced epilepsy mice

After completion of PTZ kindling, we performed the MWM test to assess the effect of 11β-HSD1 knockdown on spatial learning and memory. In the acquisition trials, the escape latency of all groups gradually decreased during the five training days. However, the epilepsy group showed significantly increased escape latency compared to the control group, while there was no statistical difference between the epilepsy group and the Con-shRNA group. Interestingly, the escape latency was significantly decreased in the 11β-HSD1-shRNA group compared to the Con-shRNA group (p = 0.043 for the 2nd, p < 0.01 for 4th and 5th day, Fig. 4C). In the probe trial, mice in the epilepsy group and the Con-shRNA group showed a decrease in the number of crossing the target quadrant and swimming velocity compared to the control group and the 11β-HSD1-shRNA group (p < 0.05, Fig. 4D–F). This data indicates that knockdown of 11β-HSD1 alleviated the impairment of spatial learning and memory induced by PTZ- kindling.

### 11β-HSD1 knockdown attenuates hippocampal neuronal damage

**Fig. 5:**
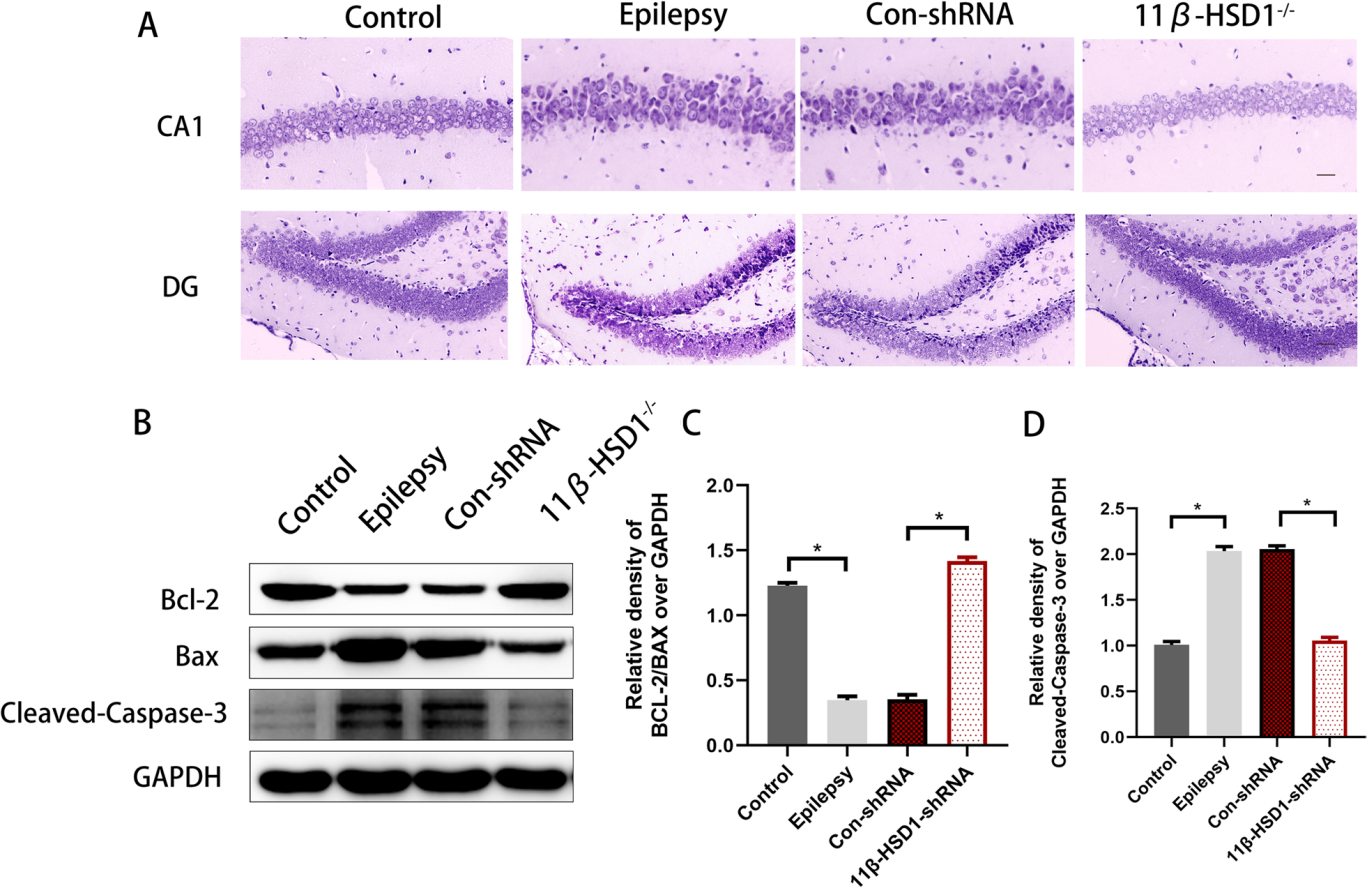
11β-HSD1 knockdown attenuated hippocampal neuronal damage and inhibited hippocampal apoptosis in the PTZ-induced epilepsy mice. **A** Representative micrographs (original magnification, × 400) of Nissl staining show that 11β-HSD1 knockdown alleviated hippocampal neuronal damage induced by PTZ kindling in CA1 and DG. **B**–**D** Western blot images show BCL-2, BAX and Cleaved Caspase-3 proteins expression (*P < 0.05, epilepsy group vs control group, Con-shRNA group vs 11β-HSD1-shRNA group). The bars indicated the mean ± SD

The hippocampus is known as a critical structure for spatial learning and memory, so we subsequently explored the effects of 11β-HSD1 knockdown on hippocampal neuronal damage induced by PTZ kindling using Nissl staining. Hippocampal CA1 and DG areas of mice in the epilepsy group exhibited considerable cell loss compared to the control group. However, the 11β-HSD1-shRNA group showed significantly attenuated hippocampal neuronal damage compared to the Con-shRNA group (Fig. 5A). The above data showed that knockdown of 11β-HSD1 ameliorated the hippocampal neuronal damage induced by PTZ kindling.

### 11β-HSD1 knockdown inhibited hippocampal apoptosis in the PTZ-induced epilepsy mice

As apoptosis is known to play a role in neuronal damage associated with epilepsy [3], we then examined molecular markers of apoptosis in the hippocampus using Western blot. The epilepsy group displayed a significantly increased ratio of Bcl-2/Bax (p < 0.05, Fig. 5B, D) and decreased expression of the apoptosis-related protein cleaved caspase-3, compared to the control group (p < 0.05, Fig. 5C, D). There were no statistical differences between the epilepsy group and the Con-shRNA group. In contrast, the 11β-HSD1-shRNA group presented significantly increased Bcl-2/Bax ratio and decreased cleaved caspase-3 level compared to the Con-shRNA group (p < 0.05, Fig. 5B–D). The above data indicated that knockdown of 11β-HSD1 inhibited hippocampal apoptosis caused by PTZ kindling.

## Discussion

In the present study, we found that 11β-HSD1 was mainly expressed in the neurons, and its expression was significantly increased in a mouse epilepsy model induced by PTZ. Overexpression of 11β-HSD1 significantly decreased the threshold voltage and increased the frequency of AP firing in primary cultured hippocampal neurons. Local knockdown of 11β-HSD1 in the hippocampus not only reduced seizure severity and kindling epileptogenesis but also alleviated the cognitive impairment of epileptic mice, and this was accompanied by reduced apoptosis and neuronal loss in the hippocampus of PTZ kindling epilepsy mice.

HPA axis activation is involved in the regulation of a variety of life activities. It is well recognised that epilepsy is closely related to the HPA axis. Human epileptic seizures, especially generalised tonic clonic seizures and complex partial seizures, which are common in temporal lobe epilepsy (TLE), result in increased activation of the HPA axis [25–27]. Similarly, in animals, even a single evoked temporal lobe seizure in healthy (non-epileptic) rodents can activate the HPA axis, significantly increasing corticosterone levels [28, 29]. However, as a critical enzyme of glucocorticoid metabolism, the expression patterns of 11β-HSD1 in epilepsy have, to date, not been explored. Since 11β-HSD1 is highly expressed in the hippocampus, a critical brain region involved in epilepsy, this study first examined the expression of 11β-HSD1 in the hippocampus of epilepsy mice. We found that 11β-HSD1 was mainly expressed in the neurons rather than astrocytes, and the expression of 11β-HSD1 was significantly upregulated in the hippocampus of PTZ-induced epileptic mice, indicating a possible association of 11β-HSD1 and development of epilepsy. This possibility was further enhanced by the results of in vitro whole-cell patch clamp recordings, which showed that the overexpression of 11β-HSD1 significantly increased the excitability of primary cultured hippocampal neurons. Based on these results, we hypothesised that inhibiting the expression of 11β-HSD1 might have an anti-epileptic effect by suppressing the excessive excitability of neurons in epilepsy.

To test this hypothesis, we developed a hippocampal11β-HSD1 knockdown model, and then assessed its impact on our epilepsy model. We found that knockdown of hippocampal 11β-HSD1 significantly decreased the severity of seizures and increased the latency of complete kindling induced by PTZ. 11β-HSD1 affects glucocorticoid metabolism by catalysing circulating inert glucocorticoid to active hormone, and so increased expression of 11β-HSD1 would be anticipated to increase hippocampal steroid activity. Therefore, knockdown of 11β-HSD1 will inhibit glucocorticoid activity. As hyper-activation of glucocorticoids can reduce seizure threshold and increase neuronal excitability in TLE [7], and potentiate neuronal injury in the hippocampus of mouse epilepsy model [30–32], knockdown of 11β-HSD1 will result in decreased epileptic activity. These results are also consistent with previous studies of controlling epilepsy through intracranial glucocorticoid intervention [33, 34] and proved the involvement of 11β-HSD1 in epileptogenesis.

Cognitive impairment is one of the major comorbidities of epilepsy [3]. Studies have shown that elevated hippocampal and neocortical 11β-HSD1 is observed during aging and causes cognitive decline and that experimental 11β-HSD1 deficiency prevents the emergence of cognitive defects associated with aging [35]. Since the expression of 11β-HSD1 was increased in the brain of epileptic mice, this may negatively impact cognition in epileptic mice. In the MWM test, we found that the escape latency of PTZ kindled mice was longer compared with the control group. Also, after the hidden platform was removed, the number of crossing the target quadrant was decreased in PTZ kindling mice, indicating that PTZ kindling impaired spatial learning and memory, which is consistent with previous studies [36, 37]. However, hippocampal knockdown of 11β-HSD1 was able to rescue these cognitive deficits. A previous study found that elevated 11β-HSD1 in adults was associated with increased incidence of brain atrophy, leading to cognitive dysfunction [38]. Furthermore, in two randomised, double-blind, placebo-controlled crossover studies, short-term administration of a nonselective 11β-HSD1 inhibitor, carbenoxolone, improved verbal fluency and memory in a small cohort of adults with type 2 diabetes [15]. Our results align with these prior studies, and suggest that inhibition of 11β-HSD1 is a promising target for treating both epilepsy and the associated cognitive impairment.

Regarding the pathological mechanisms, apoptosis plays a critical role in the pathogenesis of epilepsy. Repetitive epileptic seizures lead to neuronal apoptosis [37], and neuronal apoptosis aggravates seizures [39]. In addition, hippocampal neuronal apoptosis has been shown to contribute to impaired hippocampus-dependent cognitive function [40]. Therefore, researchers have attempted to use pharmacotherapy targeting neuronal apoptosis to improve cognitive impairment, for example caused by vascular ischemia or epilepsy [37, 41]. Prior studies have shown that 11β-HSD1 overexpression causes apoptosis in insulinoma cells [20], and inhibiting the activity of 11β-HSD1 can alleviate apoptosis in spleen cells [42]. Thus, we proposed that inhibition of 11β-HSD1 activity might protect against the hippocampal neuronal damage of PTZ kindling epileptic mice by reducing apoptosis. To test this hypothesis, we examined the neuronal damage and expression of apoptosis-related proteins. We found that knockdown of 11β-HSD1 reduced neuronal injury in hippocampal CA1 and DG areas induced by PTZ kindling, preventing the increased expression of pro-apoptosis proteins Bax and cleaved caspase-3 and the reduced expression of anti-apoptosis protein Bcl-2 in PTZ kindled epilepsy mice. These data suggest that inhibition of 11β-HSD1 activity can reduce hippocampal apoptosis in epilepsy and thus exert a neuroprotective effect against neuronal damage induced by epileptic seizures, which may subsequently contribute to the improvement of both epilepsy and the associated cognitive impairment.

## Conclusions

In this study, we demonstrated that 11β-HSD1 was expressed in hippocampal neurons and upregulated in the mouse epilepsy model induced by PTZ kindling. Plasmid-induced overexpression of 11β-HSD1 increased the excitability of primary cultured hippocampal neurons, and local knockdown of hippocampal 11β-HSD1 alleviated seizures and the associated cognitive impairment caused by PTZ, attenuated hippocampal neuronal damage and inhibited apoptotic cell death. Our results indicate that inhibition of 11β-HSD1 may be a promising strategy to treat both epilepsy and concomitant cognitive impairment.

## Data Availability

The datasets used and analysed during the current study are available from the corresponding author upon reasonable request.
